# Dietary supplementation of black cumin (*Nigella sativa*) meal in the formulation of protein‐enriched cookies, further in vivo evaluation of protein quality with physicochemical and organoleptic characterization

**DOI:** 10.1002/fsn3.4016

**Published:** 2024-07-23

**Authors:** Rizwana Batool, Rabia Ramzan, Awais Raza, Mahwash Aziz, Madiha Rohi, Adan Naeem, Wajeeha Nusrat, Aiza Razi, Bakhtawar Saleem, Wajeeha Batool, Ahmed Bilal, Babirye Khadijah

**Affiliations:** ^1^ Department of Food Science and Technology Government College Women University Faisalabad Pakistan; ^2^ College of Food Science and Technology Huazhong Agricultural University Wuhan China; ^3^ University Institute of Food Science and Technology, Faculty of Allied Health Sciences The University of Lahore Lahore Pakistan; ^4^ University Institute of Diet and Nutritional Sciences, Faculty of Allied Health Sciences The University of Lahore Lahore Pakistan; ^5^ Department of Food Science and Nutrition Islamic University in Uganda Mbale Uganda

**Keywords:** amino acid profile, black cumin (*Nigella sativa*), DBCM, flour blends, in vivo evaluation, organoleptic properties

## Abstract

*Nigella sativa* has solid historical importance as a medicinal herb and is widely used in the bakery industry. The study revealed that the defatted black cumin meal (DBCM) possesses a high nutritious composition and amino acid profile, such as Leu, Lys, Arg, Phe, and Glu. The supplementation effect of wheat flour with defatted black cumin meal (DBCM) at levels of 5%–25% was explored on the cookies' nutritional, functional, and organoleptic attributes. Moreover, the present work was conducted to assess the in vivo protein quality of defatted black cumin meal (DBCM)‐supplemented cookies using the albino rat's experimental modeling for 10 days. Rheological characterization suggested a solid elastic behavior for all cookies dough with elevation in *G*' (138,652–230,926 Pa), *G*" (34201–45,092 Pa), and G* values due to protein addition. The functional characteristics of the flour blends showed that DBCM addition significantly (*p* ≤ .05) improved oil (70.56%–165%) and water (84.83%–232.67%) absorption capacities, emulsion, and foaming stability. The physicochemical and organoleptic assessment of the cookies exposed that a 20% level of substitution of DBCM produced acceptable cookies. The net protein utilization (NPU), protein efficiency ratio (PER), true digestibility (TD), and biological value (BV) significantly differ from each other among all the supplementation diet groups with 0%–25% DBCM.

## Graphical abstract

*Nigella sativa*, with its rich historical significance as a medicinal herb, plays a crucial role in the bakery industry. This study investigated the impact of supplementing wheat flour with defatted black cumin meal (DBCM) at varying levels (5%–25%) on the nutritional, functional, and sensory characteristics of cookies. The physicochemical and organoleptic evaluations of the cookies revealed that a 20% substitution of DBCM resulted in cookies that met acceptable standards. The supplementation levels (0%–25% DBCM) exhibited significant differences in net protein utilization (NPU), protein efficiency ratio (PER), true digestibility (TD), and biological value (BV) among the various diet groups.

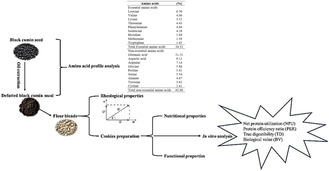

## 1 INTRODUCTION

The entire parts of black cumin (*Nigella sativa* L.) seeds are almost used for culinary and medical purposes, such as seasoning a variety of foods and treating specific ailments. In terms of potential applications, it is critical to thoroughly understand the *N. sativa* seed's composition. The content of black cumin varies depending on environmental distribution, harvest period, and agronomic procedures, as with most herbs. Total carbohydrates, oil, moisture, ash, and protein contents were found to be in the range 24.9%–40.0%, 22.0%–40.35%, 3.8%–7.0%, 20.85%–31.2%, and 3.7%–4.7%, respectively, according to some scientific studies (Kour & Gani, 2021; Trigui et al., 2021). It has a high concentration of tocopherols and other beneficial substances. According to the findings of Albakry et al. (2022), stigmasterol and β‐sitosterol are the main constituents, collectively making 65% of overall sterols. Moreover, minute quantities of lanosterol, campesterol, and D7‐avenasterol are also present. *Nigella sativa* seed is one of the most important antidiabetic plants highly recommended by traditional practitioners (Kour et al., 2021). Black cumin has been considered one of the most treasured nutrient‐rich herbs in history around the world (Bashir et al., 2021).

Butt and Sultan (2010) stated the availability of several functionally essential components such as phytosterols, PUFA, antidiabetic, antioxidants, antitumor, and other active ingredients in *N. sativa* seeds and oils lowered the risk of several diseases. Important biological activities of *N. sativa* seeds are mainly due to the carvacrol (6%–12%), p‐cymene (7%–15%), and thymoquinone (30%–48%) compounds. Black cumin's indigenous cultivar native to Bangladesh 429 has nutritionally essential sesquiterpene longifolene (1%–8%), t‐anethole (1%–4%), and 4‐terpineol (2%–7%) (Mazaheri et al., 2019). Pharmacologically, it is assumed that the most critical active elements of *N. sativa* are thymol and thymohydroquinone (Kaur et al., 2021). Additionally, it has been reported that thymoquinone effectively regulates serological and hematological activities, which help in maintaining the homeostasis of the body (Hussain & Hussain, 2016). There are appreciable quantities of polyunsaturated (48%–70%), monounsaturated (18%–29%), and saturated (12%–25%) fatty acids in black cumin seed fixed oil. It has also been reported that the most abundant quantities of monounsaturated and polyunsaturated fatty acids in black cumin seeds are oleic and linoleic acids, respectively (Hassanien et al., 2014). The health‐promoting benefits of black cumin are often attributed to its lipid fractions, which are available in the market as *Nigella sativa* oil (Hannan et al., 2021). The seeds contain an appreciable quantity of oil ranging from 22.0% to 40.35% (7). The material left after oil extraction has limited application, and defatted black cumin meal (DBCM) has not been utilized previously for value‐added product development. The macro‐ and micronutrients in DBCM can be a useful addition to wheat flour, a staple diet of the region, to combat several nutrition‐related syndromes. Furthermore, DBCM addition may serve as a tool to supplement wheat or corn flour proteins. The products made from the resulting blends would be of high protein quality and cost‐effective, too. Flour blends are vital for developing enriched products with optimal utility and added health benefits (Onwuzuruike et al., 2022).

The nutritional profile of flour blends and their functional properties are important while investigating their potential during the phase of product development. Physicochemical properties of the resultant blends affect proteins' behavior during processing, storage, cooking, and consumption. Besides nutrition, the functional properties of value‐added food components play an essential role in their successful absorption into traditional food composition (Alamu et al., 2021).

The use of DBCM as a nutrient‐dense source has the potential to be incorporated into wheat flour‐based products, such as cookies and bread. The present study was designed to explore the in vivo protein quality and nutritional and functional properties of DBCM‐based wheat flour blends for their ultimate use in cookies to combat the issue of protein energy malnutrition.

## 2 MATERIALS AND METHODS

### 2.1 Procurement of material

*Nigella sativa* seeds were obtained from BARI (Barani Agricultural Research Institute), Chakwal. The wheat flour was procured from Sunny Flour Mills, Lahore. All chemicals and reagents were purchased from Sigma‐Aldrich (Sigma‐Aldrich, Tokyo, Japan) and Merck (Merck KGaA, Darmstadt, Germany).

### 2.2 Preparation of DBCM

Extraction of black cumin oil was carried out using solvent extraction (n‐hexane) process, as described in AOCS, 2017 with Soxtec System (Model: H‐21045 Extraction Unit, Hoganas, Sweden) through continuous refluxing of solvent. Processing of the DBCM was done according to the method of Osman et al. (2015).

### 2.3 Nutritional assessment of black cumin seeds (BCS)

BCS and resultant defatted meal were analyzed for nitrogen‐free extract (NFE), protein, moisture, crude fiber, ash, and crude fat, by following the methods of Anonymous and AACC (2000). Calcium, magnesium, zinc, and iron were determined by spectrophotometer (Varian AA240, Australia), while sodium and potassium were determined by Flame Photometer‐410 and atomic absorption spectrophotometer (Albakry et al., 2022).

### 2.4 Amino acid analysis

The seed protein was acid hydrolyzed in a mini‐reaction vial with 3 mL 6 N HCl and 0.2 g of seed over 16 h at 110°C in a nitrogen environment. Following the completion of the hydrolysis, the vial contents were filtered, making a filtrate of up to 10 mL. Amino acid analysis was conducted using a JLC‐500/V amino acid analyzer (JEOL Ltd., Japan), utilizing 100 μL of this hydrolysate and 2 μL of the internal standard alpha‐amino adipic acid. The amino acid analyzer was equipped with an LCR‐6 column with 4 mm × 120 mm dimensions. After integrating the chromatographic peaks, identification and quantification of the peaks were done using the Breeze^TM^ software version 3.20. The peaks were compared with the known amino acid standards procured from the Amino Acid Standard Pierce and Rockford, Illinois. The same hydrolysis method and performic acid oxidation were used to determine the cysteine and methionine. HPLC was used to analyze the tryptophan after alkali hydrolysis (Kabir et al., 2019).

### 2.5 Wheat flour and DBCM blends

DBCM flour blends were formulated by replacing straight‐grade wheat flour with 5%, 10%, 15%, 20%, and 25% of DBCM (DBCM‐5, DBCM‐10, DBCM‐15, DBCM‐20, and DBCM‐25, respectively).

### 2.6 Functional properties of flour blends

A range of functional aspects, such as water and oil absorption capacities, emulsion, bulk density, foaming characteristics of flour blends, and DBCM, were evaluated. The flour sample (50 g) was placed in 100 mL measuring cylinder and tapped several times to a constant volume. The bulk density (g/cm^3^) was obtained by dividing the weight of flour (g) by the volume of flour (cm^3^). Each flour blend (5 g) was mixed in distilled water (25 mL) or corn oil, and then centrifugation was done at 3000 ***g*** for 25 min. Then, the supernatants were transferred, then drained the excessive moisture content at 50°C for 25 min, and samples were weighed again. The number of grams of water/corn oil bound per gram of material on a dry basis was used to calculate the water and oil absorption capacity. The method of Osman et al. (2015) was used to determine emulsifying capacity and stability.

Homogenization of the samples was done in 50 mL water for 30 s. Then, 25 mL of corn oil was added, and homogenization was done for 30 s. By dividing the volume of the emulsified layer by the volume of emulsion before centrifugation at 1000 ***g***, the emulsifying capacity was estimated. After heating samples for 15 min at 85°C, the emulsion stability was measured. In a nutshell, a blender was used to homogenize 50 mL of a 3% (w/v) dispersion of sample in distilled water for 2–3 min. Before and after whipping, the volume of foam generated was measured. The percent increase in volume due to whipping was used to calculate foaming capacity. Changes in foam volume were monitored after 60 min intervals to determine foam stability (equal to the percent of the starting foam) reported by Chandra et al. (2015).

### 2.7 Preparation of cookies

Cookies were prepared according to AACC (Method No. 10‐50D) method (Anonymous and AACC, 2000), with some modifications using wheat flour DBCM blends. In short, the components were weighed, and the creaming process (which involved blending shortening and sugar) was completed before adding the eggs. To make a homogeneous mixture, the flour blends, cocoa powder, and baking powder were also added. After that, the batter was flattened and cut with a cookie cutter. The cookies were baked in the baking oven for 15 min at 425°F. The cookies were baked, chilled, and stored in plastic bags until they were evaluated.

### 2.8 Rheological properties of dough

The controlled strain rheometer (Thermo Scientific HaakeRheoStress 1, Thermo Fisher Scientific, Schwerte, Germany) and a water bath (Phoenix II P1‐ C25P) were used for the study of the rheological behavior of doughs. A water bath was used to maintain the temperature of the analysis at 25°C. A parallel‐plate geometry (60 mm) was used with a gap of 3 mm. After the gap adjustment (3 mm), Vaseline oil was applied around the exposed surfaces of the samples during the analysis to prevent the samples from drying. The dough was rested (800 s) before measuring the oscillatory tests. In order to identify the linear viscoelastic, first of all, a strain sweep test with a 0.1–100 Pa stress range and a constant frequency of 1 Hz was performed at 25°C. Based on the obtained results, the frequency sweep test was performed by adding a stress value in the linear viscoelastic region with a frequency range of 0.1–10 Hz at 25°C. For different frequency values, the elastic modulus (*G*' [Pa]), viscous modulus (*G*" [Pa]), complex modulus, and tan *δ* (*G*"/*G*') values were obtained, and all samples were analyzed three times (Guerra‐Oliveira et al., 2021).

### 2.9 Physical and chemical analysis of cookies

Compositional analysis of bread samples was carried out to determine the moisture, ash, fat, fiber, protein, and total carbohydrate contents using the procedure outlined by AOAC (2007).

### 2.10 Organoleptic analysis

Sensory evaluation has been performed according to the method described by Ali et al. (2020). An expert sensory panel of judges was usually made up of 10 people. They were selected based on their ability to perceive tastes and aromas, differentiate between products, and accurately describe what they perceive.

### 2.11 In vivo estimation of protein quality

All the treatments, including DBCM, were chosen for in vivo assessment of the quality of protein based on the findings of the physicochemical and sensorial assessment of cookies. The 5%, 10%, 15%, 20%, and 25% of DBCM (DBCM‐5, DBCM‐10, DBCM‐15, DBCM‐20, and DBCM‐25) diets were prepared using flour from cookie samples. Table 1 shows the diet composition. The AOAC (2007) guidelines were used to formulate the diets. Weanling male albino rats (42–45 g) of 28 days old were divided into six groups based on their weights. The grouping was done using a randomized block design in such a manner that the difference in mean initial weights with ±0.5 g. There were 10 rats in each group and housed in wire‐bottom cages in order to collect feces and measure the food intake in an easy manner. The animals' room temperature was maintained at 25 ± 1°C with alternate dark and light periods of 12 h. C +ve (containing casein) was fed to one group of 10 rats. The second group was provided with a C −ve diet (without protein diet) consisting of a basal diet. Whereas the experimental diets were fed to the remaining four groups with free access to water and diet. In addition, according to Grant et al. (1995), the diets were added with minerals and vitamins to achieve the target requirements. The records of weight loss and gain, protein and food intake, and fecal and urinary outputs of rats were taken daily. A polystyrene beaker was placed over the urethra and the base of the tail was stimulated. Kjeldahl (AOAC, 2007) method was used to determine the fecal and urinary nitrogen contents from the suitable test diet groups. Protein efficiency ratio (PER), true digestibility (TD), biological value (BV), and net protein utilization (NPU) were determined by using the data obtained from the previous experiment as reported by Berardy et al. (2019). BV is the proportion of nitrogen (absorbed) that is reserved by the rats after endogenous nitrogen and urinary correction. The deceptive digestibility modified for the metabolic nitrogen (N) in fecal materials is the true digestibility (TD). The net or overall utilization of protein is determined from the TD/BV × 100.

**TABLE 1:** Composition of experimental diets fed to rats.

Diet	Ingredients of diet (g/100 g)						
	Cookies	Corn oil	Mineral mixture	Vitamin mixture	Corn starch	Casein	DBCM
C −ve	–	10	2.5	1	86.5	–	
C +ve	–	10	2.5	1	76.5	10	
DBCM	–	10	2.5	1	76.5	–	10
DBCM‐0	83.7	10	2.5	1	2.8	–	–
DBCM‐5	78.2	10	2.5	1	8.3	–	–
DBCM‐10	73.7	10	2.5	1	12.8	–	–
DBCM‐15	69.1	10	2.5	1	17.4	–	–
DBCM‐20	64.4	10	2.5	1	22.1	–	–
DBCM‐25	59.6	10	2.5	1	26.9	–	–

Abbreviations: C –ve, Diet without any protein; C +ve, Casein protein diet; DBCM, Defatted black cumin, DBCM‐0 (0%), DBCM‐5 (5%), DBCM‐10 (10%), DBCM‐15 (15%), and DBCM‐20 (20%).

### 2.12 Statistical analysis

All experimental analyses were accomplished in triplicate form. Statistics 8.1 program (Analytical Software, USA) was utilized for the statistical analyses. In the Tukey test, the *p*‐value <.05 was Tukey method was applied by using the statistical program Statistics 8.1 (Analytical Software, USA) for analysis of variance (ANOVA) and comparison of means. *p* < .05 is considered as statistically significant and *p* < .01 as highly significant.

## 3 RESULTS AND DISCUSSIONS

### 3.1 Nutritional composition of raw and defatted black cumin meal (DBCM)

The findings in Table 2 indicated the proximate composition of full‐fat and defatted black cumin meal; moisture contents were 6.29% and 5.68% in black cumin seeds (BCS) and DBCM, respectively. Protein contents increased from 23.15% to 33.37% after oil extraction, whereas fiber was 5.98% in full‐fat black cumin seeds, which increased to 9.34 ± 0.41% in DBCM. Similarly, the resultant meal was rich in ash (6.55%) compared to parental material (4.15%). The results further indicated that potassium (812 mg/100 g), calcium (575 mg/g), phosphorus (548 mg/g), and magnesium (260.33 mg/g) were dominant minerals in FFBC. Similarly, in DBCM, the respective minerals were dominant, with 20% higher values than full fat. The indigenous variety used in the present investigation was also explored by Kour and Gani (2021), who reported that it contains 6.46% (moisture), 22.80% (proteins), 31.16% (fat), and 6.03% (fiber). However, after oil extraction, DBCM had a higher amount of protein and fiber. The findings of current research agree with those reported by Trigui et al. (2021); however, they differed somewhat from those stated by Batur and Kutluay (2022).

**TABLE 2:** Nutritional profiling of black cumin seeds and defatted black cumin meal.

	Characteristics	Black cumin seeds	Black cumin meal
		(BCS)	(DBCM)
Proximate composition (%)	Moisture	6.29 ± 0.27	5.68 ± 0.25
	Crude protein	23.15 ± 1.01	33.37 ± 1.45
	Crude fat	31.52 ± 1.37	0.49 ± 0.02
	Crude fiber	5.98 ± 0.26	9.34 ± 0.41
	Ash	4.15 ± 0.18	6.55 ± 0.29
	Nitrogen‐free extract	28.99 ± 1.26	44.57 ± 1.94
Minerals (mg/100 g)	Potassium	812.00 ± 35.39	965.00 ± 42.06
	Calcium	575.00 ± 25.06	691.00 ± 30.12
	Phosphorus	548.00 ± 23.89	656.00 ± 28.59
	Magnesium	260.00 ± 11.33	311.00 ± 13.56
	Sodium	17.54 ± 0.76	20.85 ± 0.91
	Iron	8.95 ± 0.39	11.03 ± 0.48
	Manganese	8.43 ± 0.37	10.12 ± 0.44
	Zinc	6.43 ± 0.28	7.57 ± 0.33
	Copper	2.59 ± 0.11	3.09 ± 0.13

### 3.2 Amino acid profile

**FIGURE 1:**
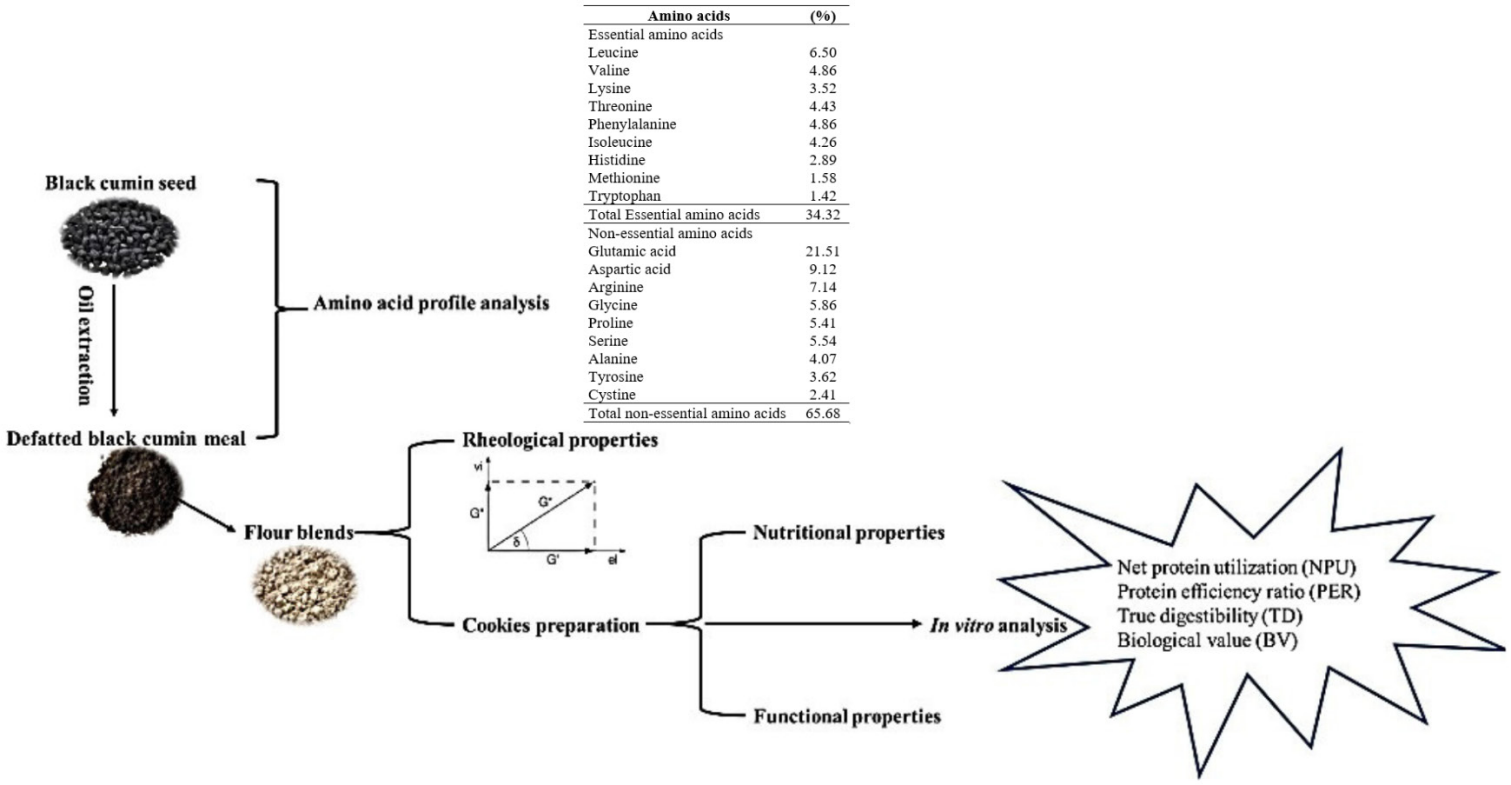
Graphical Abstract.

The physiological characteristics of proteins are defined by the amount, properties, and distribution of essential amino acids in the finished product (Mortuza & Tzena, 2009). The composition of amino acids in black cumin seeds, determined by the amino acid analyzer, is expressed in Table 3. Protein composition presented the presence of 18 amino acids having 9 essential amino acids. The amino acid with maximum content was glutamic acid (21.51%), followed by aspartic acid (9.12%), arginine (7.14%), glycine (5.86%), and leucine (6.5%) Figure 1.

**TABLE 3:** Amino acid profile of black cumin meal.

Amino acids	(%)
*Essential amino acids*	
Leucine	6.50
Valine	4.86
Lysine	3.52
Threonine	4.43
Phenylalanine	4.86
Isoleucine	4.26
Histidine	2.89
Methionine	1.58
Tryptophan	1.42
Total Essential amino acids	34.32
*Nonessential amino acids*	
Glutamic acid	21.51
Aspartic acid	9.12
Arginine	7.14
Glycine	5.86
Proline	5.41
Serine	5.54
Alanine	4.07
Tyrosine	3.62
Cystine	2.41
Total nonessential amino acids	65.68

This 54% of total amino acids is constituted by the primary amino acids in the black cumin seed protein. Similar findings were stated by Kabir et al. (2019), depicting glutamic acid as the most sample amino acid in black cumin seed. Methionine (1.58%) and tryptophan (1.42%), the essential amino acids, were the minor ones. Kabir et al. (2019) also reported tryptophan as the lowest amino acid in black cumin cake protein, similar to the findings of the current study. Previously, Zaky et al. (2021) also established that Asp, Arg, and Glu exist in black cumin seeds as major amino acids, whereas Met and Cys as minor amino acids. It could be deduced from the current study that an appreciable amount of nutrients is present in black cumin seeds and can be used as food supplements and might be a beneficial health source.

The presence of essential amino acids makes black cumin seeds a valuable nutritional source and is in accordance with the standards of FAO and WHO (Shaker Hassan et al., 2011). In another study, it was declared that black cumin is abundant in glutamic acid compared to arginine. It was reported that the cumin cake protein contains a maximum quantity of aromatic amino acids (Phe and Tyr) with chemical scores of 122. Furthermore, essential amino acids have exceptional prospects as food antioxidants (Sohaib et al., 2017).

### 3.3 Functional and proximate characterization of flour blends

**FIGURE 2:**
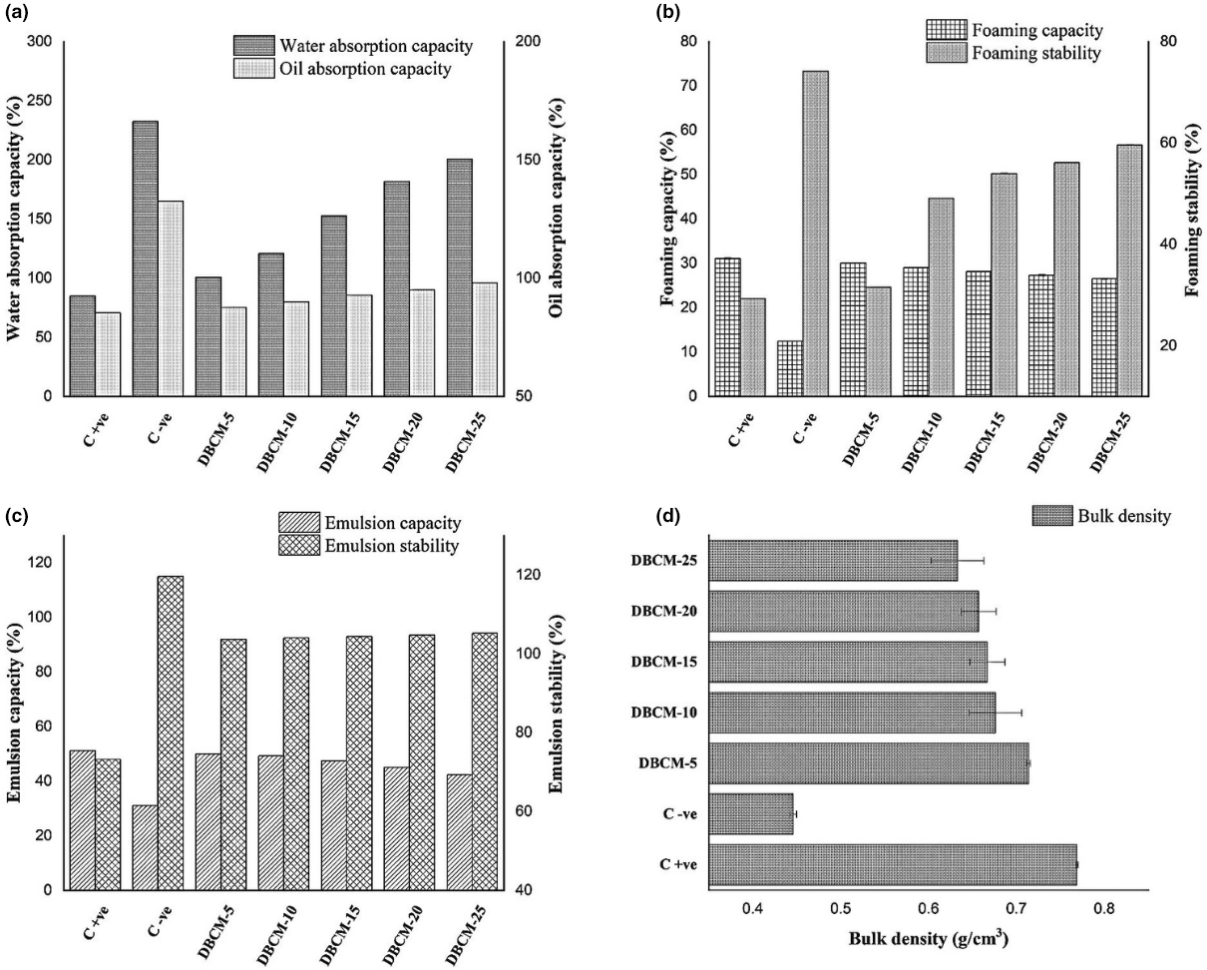
Effect of partial replacement of wheat flour with DBCM on functional properties of flour blends. (a) Water and oil absorption capacity; observed increasing tendency for these traits. (b) Foaming capacity and foaming stability; foaming capacity of flour blends decreased, however, increasing tendency was observed for foaming stability. (c) Emulsion capacity and stability; marked decrease in the emulsion capacity, however, emulsion stability increased with increment of black cumin meal (DBCM). (d) Bulk density; wheat flour was partially replaced with defatted black cumin meal (DBCM) and observed decreasing trend. Bars signify the means of triplicate ± SD.

The DBCM addition caused a decrease in the bulk density of the flour blends, that is, from 0.71 to 0.63 g/cm^3^ (Figure 2d). Results for water absorption capacity (WAC) showed that DBCM has a higher capacity to absorb water 232.67% as compared to control (84.83%). Likewise, oil absorption capacity varied from 70.56% to 165% in different flour blends (Figure 2a). The emulsion capacity of straight‐grade flour decreased gradually with the addition of DBCM (Figure 2c). The means for emulsion stability demonstrated maximum values (100.00%) in the flour blend containing 20% DBCM, whereas the minimum (48.02%) in 100% straight‐grade flour. Foaming capacity was significantly affected among different treatments. The highest foaming capacity was noted in the case of straight‐grade flour, which decreased with the addition of DBCM. However, the results of foaming stability exhibited an opposite trend (Figure 2b).

The functional properties of flour blends are essential when describing their potential food applications (Zaky et al., 2021). The bulk density of flour blends depends on the interactive effect of some integrated factors, including the size of particles, interparticle forces, and strength of contact points. In the present case, bulk density decreased with a gradual increase in DBCM (Okwunodulu et al., 2022). The interaction of the protein with macro‐ and microconstituents of food significantly influences the water‐binding characteristics. The lower water absorption capacity of straight‐grade flour is due to less availability of polar amino acids (Adanse et al., 2020).

The higher level of carbohydrates in wheat flour adversely affected the emulsion capacity (Kabir et al., 2019). The stability of emulsion of DBCM added flour blends was higher in comparison to control. Due to the high emulsion stability of DBCM, it can be applied as an ingredient in bakery products. It is also evident that, in contrast to the foaming capacity, the foaming stability increases by increasing the level of DBCM in the flour blends. The high values for foaming stability showed hydrated foams while a decrease in foaming stability could possibly be due to protein denaturation.

Therefore, wheat flour was replaced up to 25% level with that of DBCM to formulate various composite blends. The moisture content of different flour blends decreased with DBCM addition (Table 4). In contrast, protein and fiber contents increased from 10.85% to 16.16% and 0.43% to 2.66%, respectively. The ash contents of various blends increased significantly with DBCM addition to straight‐grade flour (Table 4). The nitrogen‐free extract representing carbohydrate fractions was found to be highest in control while the lowest in DBCM‐25. The enhancement in protein, fat, fiber, and ash in resultant blends was due to a higher amount of DBCM (Kour & Gani, 2021).

**TABLE 4:** Proximate composition (%) of flour blends.

Treatments	Moisture	Protein	Fiber	Fat	Ash	NFE
Control	11.95 ± 0.32a	10.85 ± 0.29e	0.43 ± 0.01f	0.88 ± 0.02a	0.55 ± 0.01f	75.34 ± 1.99a
DBCM‐5	11.38 ± 0.41ab	11.73 ± 0.42e	0.85 ± 0.03e	0.86 ± 0.03ab	0.95 ± 0.03e	74.23 ± 2.68a
DBCM‐10	11.08 ± 0.40bc	12.83 ± 0.46d	1.32 ± 0.05d	0.84 ± 0.03abc	1.28 ± 0.05d	72.65 ± 2.62ab
DBCM‐15	10.80 ± 0.60bcd	13.94 ± 0.78c	1.77 ± 0.10c	0.82 ± 0.05abc	1.65 ± 0.09c	71.02 ± 3.95ab
DBCM‐20	10.49 ± 0.55cd	15.04 ± 0.78b	2.21 ± 0.11b	0.80 ± 0.04bc	1.93 ± 0.10b	69.54 ± 3.61ab
DBCM‐25	10.16 ± 0.44d	16.16 ± 0.70a	2.66 ± 0.12a	0.78 ± 0.03c	2.27 ± 0.10a	67.97 ± 2.96b

*Note*: Control = 100% straight‐grade flour (SGF); DBCM‐5, DBCM‐10, DBCM‐15, DBCM‐20, and DBCM‐25 represent 5%, 10%, 15%, 20%, and 25% replacement of SGF with defatted black cumin meal (DBCM), respectively. Mean values (*n* = 3) followed by the same letter in the same column are not significantly different (*p* < .05).

### 3.4 Rheological properties of cookies dough

Rheological properties of dough largely depend upon the presence of constituents added, that is, starch, protein, as well as water. Moreover, their quantity also has importance, which consequently affects the handling of the dough. The handling of dough is difficult if it is too soft or too hard. Hence, the dough should be cohesive enough to bind different ingredients together during various processing stages, and dough viscoelasticity is important in clean separation when the dough is molded (Gujral et al., 2003). Results for rheological characteristics of dough are presented in Table 5. It is depicted from the results that elastic moduli (*G*') exhibited a higher value than viscous moduli (*G*") for all the samples throughout the range of frequency, which proposes an elasticity behavior in all groups of the cookie doughs studied.

**TABLE 5:** Dynamic oscillatory test results of the dough for protein‐enriched cookies prepared from flour blends of wheat and defatted black cumin seeds.

Flour blends	*G*' (Pa)	*G*"(Pa)	G*	Tan*δ*
100–0	138,652^e^	34,201^e^	147,281^e^	0.25^a^
95–5	150,321^d^	38,093^d^	163,402^d^	0.24^b^
90–10	182,150^c^	40,921^c^	190,213^c^	0.22^c^
85–15	200,233^b^	42,318^b^	223,410^b^	0.21^d^
80–20	230,926 ^a^	45,092^a^	240,926^a^	0.19^e^

*Note*: *G*': elastic moduli; *G*": viscous moduli; G*: complex moduli; tan δ: tangent delta. Mean values (*n* = 3) followed by the same letter in the same column are not significantly different (*p* < .05).

Furthermore, the protein elevated G*, *G*', and *G*" values and declined tanδ. The current study's findings align with the results reported by Inglett et al. (2015) when flour with higher protein content was used to substitute wheat flour in cookie dough. Furthermore, similar observations were reported by Mancebo et al. (2016) during their research on evaluating rice‐based starch mixed with protein to prepare gluten‐free sugar‐snap cookies.

### 3.5 Textural characterization and physicochemical composition of cookies

**FIGURE 3:**
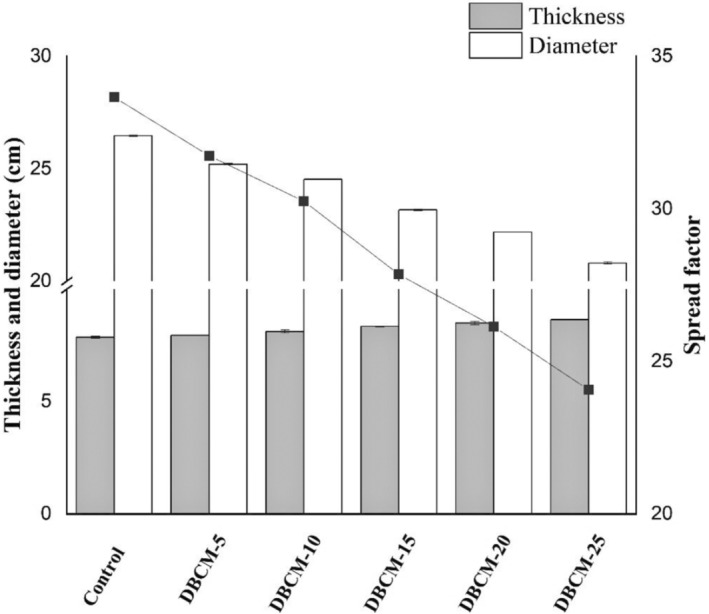
Textural properties of cookies. The bar represents the thickness and diameter, ■ with line represents the spread factor values. Bars signify the means of triplicate ± SD.

The results of the cookies evaluation are shown in Figure 3. The control exhibited a minimum thickness of 7.86 cm, while a maximum (8.64 cm) was observed in cookies with 25% DBCM. Data pertaining to diameter and spread ratio of cookies indicated that the addition of DBCM declined the attribute significantly.

The chemical composition of cookies showed that the addition of DBCM resulted in a marked increase in protein, fiber, and ash contents, that is, 6.07 to 8.70, 0.32 to 1.44, and 0.35 to 1.23%, respectively, while NFE reduced significantly from 65.62% to 61.14% (Table 5). However, moisture contents did not vary significantly and were found in the range 3.02%–5.48% (Table 6). According to the author, exploring the functional properties of wheat flour DBCM blends is tested for the first time to prepare protein‐enriched cookies. The choice of cocoa‐based cookies was to mask the slight blackish tonality of DBCM. Type and wheat quality, gluten development, water‐ingredient interaction, and the use of emulsifiers are all aspects that influence the physical characteristics of cookies. Furthermore, cookies' size and spread potential are controlled by flour particle size and moisture concentration (Korese et al., 2021). In general, replacing regular shortening with vegetable oil enhances spreadability, whereas adding bran reduces spreadability (Culetu et al., 2021). Similarly, improvements in chemical characteristics of bread like protein, fiber, and ash contents are attributed to a higher number of components in the BC, as well as the mixed flour of BC concentration influenced the bread hardness and chewiness and more compact texture in the bread samples (Ghadarloo et al., 2023).

**TABLE 6:** Physicochemical characteristics of cookies.

Moisture	Protein	Fat	Fiber	Ash	NFE	Moisture
Control	3.21 ± 0.16^f^	6.47 ± 0.06^a^	24.44 ± 0.04^f^	0.32 ± 0.02^f^	0.35 ± 0.03^f^	65.62 ± 0.51
DBCM‐5	3.69 ± 0.12^e^	6.02 ± 0.04^b^	25.82 ± 0.06^e^	0.53 ± 0.03^e^	0.55 ± 0.06^e^	64.39 ± 0.64
DBCM‐10	4.03 ± 0.12^d^	5.83 ± 0.02^c^	27.64 ± 0.01^d^	0.76 ± 0.01^d^	0.72 ± 0.05^d^	63.93 ± 0.21
DBCM‐15	4.79 ± 0.18^c^	5.68 ± 0.08^d^	28.78 ± 0.02^c^	0.99 ± 0.05^c^	0.91 ± 0.01^c^	62.85 ± 0.33
DBCM‐20	5.02 ± 0.15^b^	5.21 ± 0.03^e^	29.67 ± 0.07^b^	1.22 ± 0.08^b^	1.06 ± 0.04^b^	61.82 ± 0.94
DBCM‐25	5.48 ± 0.21^a^	5.02 ± 0.02^f^	30.41 ± 0.03^a^	1.44 ± 0.03^a^	1.23 ± 0.05^a^	61.14 ± 0.30

*Note*: Control = 100% straight‐grade flour (SGF); DBCM‐5, DBCM‐10, DBCM‐15, DBCM‐20, and DBCM‐25 represented 5%, 10%, 15%, 20%, and 25% replacement of SGF with defatted black cumin meal (DBCM), respectively. Values have been expressed as mean ± SD (standard deviation) that were analyzed individually in triplicate. The values presented in a column with different superscripts are significantly different (*p* < .05).

### 3.6 Organoleptic properties of cookies

**FIGURE 4:**
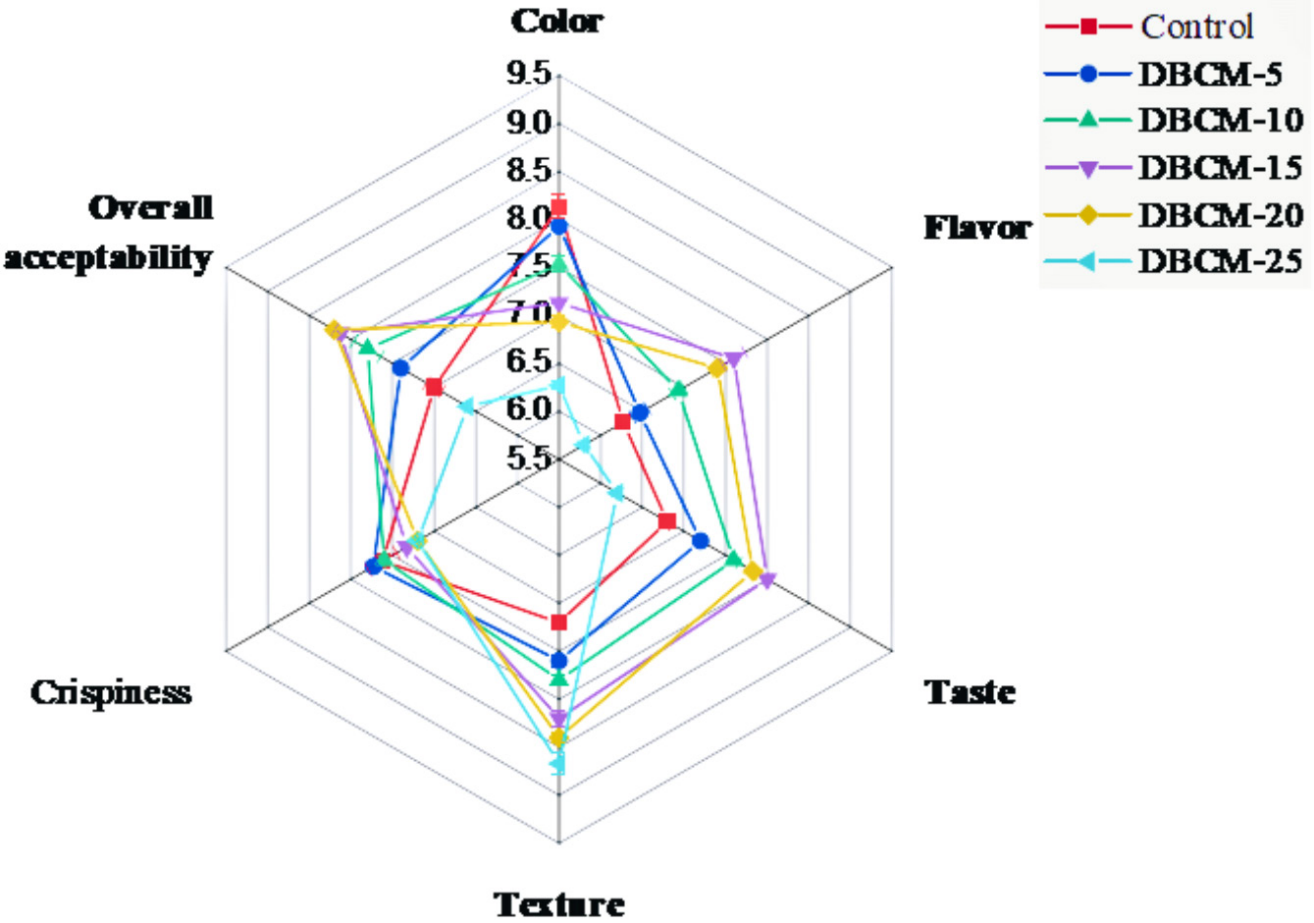
Mean scores for organoleptic traits of cookies. Control = 100% straight‐grade flour (SGF); DBCM‐5, DBCM‐10, DBCM‐15, DBCM‐20, and DBCM‐25 represented 5%, 10%, 15%, 20%, and 25% replacement of SGF with defatted black cumin meal (DBCM), respectively. The values were represented as average values ± SD.

Results regarding sensory evaluation indicated that the addition of DBCM significantly affected color, taste, flavor, crispiness, texture, and overall acceptability (Figure 4). Color scores decreased with the addition of DBCM from 8.13 (Control) to 6.27 (25% DBCM). The addition of DBCM up to 15% improves the flavor and taste while further addition results in decreasing trend in scores. The texture of protein‐enriched cookies improved with a gradual increment of DBCM; the maximum score was recorded at 25% DBCM, that is, 8.67, compared to 7.20 for control. However, the crispiness of the cookies differed significantly; the maximum score was recorded at 5% DBCM, which was statistically at par with control and 10% DBCM.

Results regarding overall acceptability revealed that 15 and 20% DBCM were rated better. On the contrary, cookies made from 25% DBCM did not get a better response from the panelists, with a mean score of 6.60 (Figure 4).

The color quality of the cookies decreased with a gradual increase in DBCM, which is masked by adding up cocoa, while texture improved, and maximum scores were assigned to 25% DBCM‐supplemented cookies. The rest of the parameters behaved differently. Overall, adding 15% and 20% DBCM was rated better for flavor, taste, and overall acceptability. Conclusively, results indicate the potential of DBCM to be incorporated in cookies to cope with protein energy malnutrition among the people at risk. Moreover, higher ash and fiber contents in resultant cookies are also a healthy sign to improve the intake of macronutrients in the vulnerable segments of developing economies. These results were in accordance with the sensory evaluation results of Ghadarloo et al. (2023), Nezamdoost‐ sani et al. (2018), and Osman et al. (2015).

### 3.7 In vivo protein quality characterization

The cookies prepared with 0, 5, 10, 15, 20, and 25% DBMC were analyzed by physicochemical as well as sensorial evaluation, with three control groups (DBMC, casein, and nonprotein diet) exposed to evaluate the quality of protein by a rat bioassay. The findings of the feeding trials on rats, executed for the evaluation of the quality of protein of cookies, as compared to casein and DBMC, are depicted in Table 7. These findings provide the information that DBCM addition improves the nutritional value of cookies. The lowest body weight gain (16.50 g) was observed in rats fed on C −ve diet and is considerably different (*p* < .05) from the DBMC‐based supplemented feed and the casein diet (C +ve). However, nonsignificant has been observed among the rat group for daily feed intakes (Table 7).

**TABLE 7:** In vivo protein quality of experimental diets.

Diet	Feed intake (g)*	Weight gain (g)*	NPU (%)	TD (%)	BV (%)	PER	FER	NPR
C −ve	11.62^i^	−10.50^i^	–	–	–	–	–	–
C +ve	12.84^a^	38.60^a^	76.92^a^	82.46^a^	93.34^a^	3.00^a^	1.99^a^	0.16^a^
DBCM	12.75^b^	34.12^b^	66.01^b^	76.11^b^	84.10^b^	2.67^b^	1.93^b^	0.19^b^
DBCM‐0	12.15^h^	22.13^h^	48.09^h^	52.15^h^	63.29^h^	1.82^h^	2.17^h^	0.25^h^
DBCM‐5	12.27^g^	24.42^g^	51.54^g^	59.35^g^	66.62^g^	1.99^g^	2.11^g^	0.24^g^
DBCM‐10	12.43^f^	27.98^f^	54.32^f^	61.09^f^	69.83^f^	2.25^f^	1.94^f^	0.23^f^
DBCM‐15	12.57^e^	29.27^e^	58.42^e^	65.87^e^	72.61^e^	2.32^e^	2.00^e^	0.22^e^
DBCM‐20	12.68^d^	31.72^d^	60.21^d^	69.26^d^	75.79^d^	2.50^d^	1.90^d^	0.21^d^
DBCM‐25	12.71^c^	32.13^c^	62.91^c^	71.65^c^	81.02^c^	2.52	1.96^c^	0.20^c^

*Note*: Values have been expressed as mean ± SD (standard deviation) that were analyzed individually in triplicate. The values presented in a column with different superscripts are significantly different (*p* < .05).

Abbreviations: BV, Biological Value; C +ve, casein protein diet; C −ve, diet without any protein; DBCM, defatted black cumin; FER, Feed Efficiency Ratio (NPU/Weight gain); NPR, Net Protein Ratio (NPU/Feed intake); NPU, Net Protein Utilization; PER, Protein Efficiency Ratio (Weight gain/Feed intake); TD, True Digestibility.

* Represent feed intake and weight gain of group of rats (10 each/10 days).

The DBCM, which has essential amino acid balance, and supplementation in cookies, increased the overall amino acid profile of the cookies, which ultimately helped to increase the body weight of the rats as compared to the control negative group. It has been observed that the feeding of diets of DBMC‐supplemented cookies (0%–25%) shows a significant (*p* < .05) effect on NPU, PER, TD, and BV than did the C +ve and C −ve diets. In brief, an overall increasing trend detected TD and BV directly. Similarly, the derived value for NPU also increased with the rise in DBMC level in cookies. However, the rat group of C −ve has low NPU, BV, and TD due to the poor protein quality diet. Cookies with 25% DBMC were nutritionally comparable with casein‐based diets, which directed that the malnutrition problem can be combated by using value‐added products of high protein wheat germ. Hence, the parameters of protein quality change due to the presence of essential amino acid content in DBMC protein. From the results, it can be concluded that the nutritionally rich cookies can be formulated by supplementing DBMC flour at 5%–25% levels in wheat flour, with increased protein, Ca, K, Fe, leucine, and glutamic acid contents.

## 4 CONCLUSIONS

It is deduced that DBCM is a promising and wholesome entity in composite flour technology to develop protein‐enriched products. The inferences drawn from in vivo protein quality and nutritional and functional properties of wheat‐defatted black cumin blends are that partial replacement of wheat flour with DBCM is feasible and provides a better protein profile to the vulnerable segment. DBCM as a cheaper source of protein and other allied micronutrients can be used as an intervention to alleviate protein energy malnutrition in developing countries.

## AUTHOR CONTRIBUTIONS

**Rizwana Batool:** Writing – original draft (equal). **Rabia Ramzan:** Data curation (equal); methodology (equal). **Awais Raza:** Formal analysis (equal); investigation (equal). **Mahwash Aziz:** Investigation (equal). **Madiha Rohi:** Writing – review and editing (equal). **Adan Naeem:** Validation (equal). **Wajeeha Nusrat:** Visualization (equal). **Aiza Razi:** Methodology (equal). **Bakhtawar Saleem:** Investigation (equal). **Wajeeha Batool:** Visualization (equal). **Ahmed Bilal:** Formal analysis (equal). **Babirye Khadijah:** Supervision (equal).

## ACKNOWLEDGEMENTS

The authors are gratefully thanking the Government College University for Women for their support.

## FUNDING INFORMATION

There was no funding for this paper.

## CONFLICT OF INTEREST STATEMENT

No potential conflict of interest was reported by the author(s).

## CONSENT FOR PUBLICATION

All authors agreed to publish the manuscript.

## Data Availability

The authors confirm that data supporting the findings of this study are available within the article.
